# Progress and Opportunities for Strengthening Global Health Security

**DOI:** 10.3201/eid2313.171758

**Published:** 2017-12

**Authors:** Frederick J. Angulo, Cynthia H. Cassell, Jordan W. Tappero, Rebecca E. Bunnell

**Affiliations:** Centers for Disease Control and Prevention, Atlanta, Georgia, USA

**Keywords:** global health security, global health protection, public health, emergency response, international health regulations, progress, opportunities

In today’s interconnected world, an infectious disease outbreak that is not rapidly detected and controlled at its source can become a costly global health threat, both in lives lost and economic turmoil (*1**,**2*). Every year, thousands of outbreaks occur worldwide, many of which involve pathogens with pandemic potential. Since 2009, the World Health Organization (WHO) has declared public health emergencies of international concern for outbreaks of influenza A(H1N1) in 2009, Ebola in West Africa in 2014, and Zika in the Americas in 2015 (*2*). In 2007, the International Health Regulations 2005 (IHR 2005) entered into force, and all 196 state parties were legally bound to implement the core capacity required under the regulations. However, in 2014, almost two thirds of member states reported not being in compliance (*3*). To accelerate progress toward IHR 2005 compliance, the Global Health Security Agenda (GHSA) was launched by 29 countries, WHO, the Food and Agriculture Organization of the United Nations, and the World Organisation for Animal Health in 2014 and now includes >60 nations (*4*).

Also in 2014, the US government provided $6 billion in emergency funding to end the Ebola epidemic in West Africa and to begin the implementation of GHSA as an initial 5-year initiative. The Centers for Disease Control and Prevention (CDC) received $1.8 billion of these funds to contain Ebola in West Africa; these efforts included assistance for countries at risk for introduction of Ebola and support for work with partners to enhance global health security through GHSA implementation. As the need for action to rapidly control outbreaks and epidemics is increasingly recognized, it is useful to take stock of accomplishments and persisting gaps in global health security.

This special supplement of *Emerging Infectious Diseases* on global health security presents an inventory of key efforts by CDC, in collaboration with many partners, to foster health security worldwide, especially by strengthening national public health core capacities. This supplement begins with several summary articles and then provides specific examples of global health security improvements in articles organized by sections entitled Prevent, Detect, and Respond.

Tappero et al. summarize selected CDC contributions to enhancing global health security from 1980 through 2017 and describe how expanded efforts under GHSA have been built on a framework created by the long history of CDC for capacity building efforts in selected partner countries (*4**,**5*). Another keynote article in the supplement describes how, after the 2014–2016 West Africa Ebola outbreak, Liberia, Sierra Leone, and Guinea have made substantial progress toward IHR 2005 implementation (*6*). To ensure credibility in the international effort to strengthen public health capacities for IHR 2005 compliance, WHO has developed and implemented the WHO Joint External Evaluation (JEE) tool (*7*). The JEE process is a vital independent global health security monitoring tool that through October 2017 has been implemented in 58 countries and is documenting both the advances toward, and gaps remaining, in national capacities for prevention, detection and control of public health threats (*5**,**7*).

## Prevent

Preventing the emergence and spread of infectious disease threats requires prevention and control of antimicrobial resistance, zoonotic diseases, vaccine-preventable diseases (VPDs), and their potential spread across international borders. Global efforts aimed at building national capacities for IHR compliance are complemented by targeted disease and country-specific efforts. For example, the Enhanced Gonococcal Antimicrobial Surveillance Program aims to inform country-specific treatment guidelines and enhance prevention and control efforts (*8*), and the President’s Malaria Initiative’s collaboration with the Antimalarial Resistance Monitoring in Africa Network supports the early detection of *Plasmodium falciparum* resistance to facilitate appropriate interventions (*9*).

Zoonoses prevention and control programs use a One Health approach with multisectoral collaboration between human and animal health. Several countries have conducted One Health prioritization exercises to identify their major zoonotic diseases (*10*), a critical step in efforts to control endemic zoonotic diseases (*11*). Ethiopia, the Democratic Republic of the Congo, and Georgia are all developing successful integrated zoonotic prevention and control programs (*12*).

Maintaining high population-wide vaccine coverage is a crucial prevention activity, particularly for VPD health security threats. For example, the failure to sustain high vaccine coverage led to a nationwide measles epidemic in Mongolia, a country previously verified as measles-free (*13*). VPD strategies include data improvement teams, which visit district health facilities and can result in improved vaccine administration data through evaluations and training efforts (*14*). Another approach for VPD is the Latin American Pertussis Project, a collaboration among 6 countries to address pertussis, a poorly controlled VPD in the region (*15*). Finally for antimicrobial resistance, zoonotic diseases, VPDs, and other public health threats, border health efforts aimed at preventing spread of communicable diseases across international boundaries are essential and have been used successfully (*16**,**17*).

## Detect

Global health security relies on all countries having >3 capacities: 1) an adequate national public health laboratory capacity to safely transport and accurately evaluate biologic specimens with appropriate diagnostic testing methods, 2) a sustained and timely public health surveillance system, and 3) a trained competent workforce to conduct essential outbreak investigations. Although rapid laboratory confirmation of public health threats is a complex endeavor, requiring long-term technical assistance and major resources, many countries are advancing key components. For example, Ghana conducted a national public health laboratory system assessment in support of the Second Year of Life initiative for sustaining adequate vaccine coverage through 24 months of age and for monitoring GHSA-sponsored public health laboratory enhancement efforts (*18*). South Korea is enhancing its public health laboratory to meet the standards of the US Laboratory Response Network, which will facilitate the ability of this country to rapidly determine the etiology of most public emergencies (*19*).

Surveillance is the cornerstone for rapidly detecting public health threats. The WHO Early Warning Alert and Response Network is a major tool for conducting public health surveillance in humanitarian emergencies, including in refugee and displaced person camps (*20*). Other vital global health security assets are CDC regional Global Disease Detection centers in 10 countries that have provided novel public health surveillance and informatics contributions alongside laboratory research since 2001 (*21*). Other disease- and country-specific efforts have also informed best practices, including enhancing anthrax surveillance programs in anthrax-endemic countries (*22*) and use of alternative surveillance approaches, such as burial permit reviews, to describe cholera mortality rates in Tanzania during a 2016 epidemic (*23*).

Rapid detection of public health emergencies also requires an adequate public health workforce, particularly trained field epidemiologists, who can conduct timely and appropriate field investigations. The CDC international 2-year Field Epidemiology Training Program (FETP) began 35 years ago and has established 65 FETP programs in 90 countries, with >3,900 graduates of the 2-year field epidemiologist training (*24*). To meet the global health need for more trained field epidemiologists, particularly at the district level, training has been expanded in many countries to include a 3-month FETP Frontline program (*25*). In 2014–2016, a total of 24 new FETP Frontline programs were initiated with >1,860 participants (*4*).

## Respond

Efficiently responding to public health emergencies is essential for preventing further disease spread and controlling outbreaks at their source. Outbreak responses worldwide have demonstrated the need for a structured incident management system, which is a critical component for a highly functional and efficient emergency operation center. Many countries, particularly GHSA partner countries, have enhanced their emergency response capacity by establishing emergency operation centers with a strong incident management system foundation (*26**,**27*). Complex humanitarian emergencies frequently involve the most difficult settings, including fragile states and areas of conflict, and recent case studies illustrate the difficulty of supporting a sustained response in such settings (*28*). After the 2014–2016 West Africa Ebola epidemic, CDC established the Global Rapid Response Team (GRRT) to ensure a ready force of trained responders. In the first 16 months, GRRT members deployed 291 times to 35 countries (*29*). Medical countermeasures, which are medical interventions aimed at controlling public health emergencies, can be essential for rapid response and containment and include using vaccination during outbreaks of cholera, typhoid, yellow fever, and Ebola virus disease (*30*).

## Conclusions

Global health security relies on IHR compliance by all countries and, as such, remains an unfinished journey (*31*). Although much has been accomplished through the first years of GHSA implementation, JEEs around the world highlight numerous prevent, detect, and respond capabilities that still need strengthening. Also lacking is an evidence base of the most effective, timely, and cost-effective approaches to building national capacities for IHR 2005 compliance. As countries and partners continue their work to build health security capabilities, there will be useful opportunities to evaluate different implementation strategies and to document the impact of newly acquired capacities. Continuing this work and thereby sustaining this momentum toward IHR 2005 compliance is critical for protecting Americans and other persons worldwide.
